# Upregulated Linc01836 in Serum Promisingly Serving as a Diagnostic and Prognostic Biomarker for Colorectal Cancer

**DOI:** 10.3389/fphar.2022.840391

**Published:** 2022-03-18

**Authors:** Lei Shen, Wei Zong, Wei Feng, Erlin Chen, Shuo Ma, Jie Yuan, Guihua Wang, Xinliang Gu, Xianjuan Shen, Shaoqing Ju

**Affiliations:** ^1^ Department of Laboratory Medicine, Affiliated Hospital of Nantong University, Nantong, China; ^2^ School of Medicine, Nantong University, Nantong, China; ^3^ Research Center of Clinical Medicine, Affiliated Hospital of Nantong University, Nantong, China; ^4^ Department of General Surgery, Affiliated Hospital of Nantong University, Nantong, China

**Keywords:** colorectal cancer, long noncoding RNA, Linc01836, diagnosis, biomarker

## Abstract

**Objectives:** Colorectal cancer (CRC) is a common carcinoma of the gastrointestinal tract with high incidence and mortality worldwide. Studies have shown that long noncoding RNAs (lncRNAs) play important roles in CRC. Our purpose is to investigate the potential of serum Linc01836 as a diagnostic and prognostic marker in CRC.

**Methods:** We evaluated the expression of Linc01836 via quantitative real-time polymerase chain reaction (qRT-PCR). The serum CEA, CA19-9, Cyfra21-1, and CA72-4 concentrations were measured by Architect I4000 SR. Receiver operating characteristic (ROC) curves were plotted to estimate the diagnostic value in CRC. Relationship between serum Linc01836 expression and clinicopathological characteristics of CRC cases was analyzed via chi-square test. The underlying mechanism of Linc01836 on the development and prognosis in CRC was predicted by bioinformatic analysis.

**Results:** The method of qRT-PCR for Linc01836 detection was confirmed with high precision and specificity. Serum Linc01836 expression in CRC patients was significantly higher than that in healthy donors (*p* < 0.0001) and benign patients (*p* < 0.0001), and declined after resection (*p* < 0.01). High expression of Linc01836 was associated with histological stage (*p* = 0.002) and lymph node metastasis (*p* = 0.006). In addition, serum Linc01836 could effectively differentiate CRC patients from the healthy folks, with favorable area under the curve (AUC) of 0.809 (95% CI: 0.757–0.861, *p* < 0.001). What is more, the combination of serum Linc01836, CEA, and Cyfra21-1 could improve diagnostic sensitivity (92.0%). Linc01836 was averagely located in the nucleus and cytoplasm, suggesting that it might participate in CRC progression and prognosis through the crosstalk among lncRNAs, miRNAs, and mRNAs.

**Conclusion:** Linc01836 may serve as a valuable noninvasive biomarker for population screening, early detection, and clinical surveillance of CRC.

## Introduction

Colorectal cancer (CRC), one of the most common digestive tract malignancies, threatens many people’s lives worldwide (Mattiuzzi et al., 2019; Siegel et al., 2021). Due to the lack of early symptoms and the limitations of diagnostic methods, most patients are found in advanced stages, and the 5-year survival rate decreases from 90% in patients at the early stage to 15% at the advanced stage (Danese et al., 2019; Chao et al., 2021). Since the frequently used CRC tumor markers like carcino-embryonic antigen (CEA), carbohydrate antigen 19-9 (CA19-9), and carbohydrate antigen 50 (CA50) do not possess comparatively satisfactory sensitivity or specificity in early screening, it is necessary to explore new biomarkers for early and effective diagnosis of CRC (Lech et al., 2016).

Long noncoding RNA (lncRNA) is a kind of noncoding RNA (ncRNA) with more than 200 nucleotides in length and without protein-coding potential. As a specific type of lncRNA, long intergenic noncoding RNA (lincRNA) does not intersect with any protein-coding locus (Peng et al., 2017). Numerous studies have illustrated that dysregulated lncRNAs have been verified to be associated with tumorigenesis, metastasis, and outcomes of various malignancies, which indicates the important utility of lncRNAs as diagnostic or prognostic tumor markers (Yu et al., 2017; Xu et al., 2018, Yu et al., 2017). Ectopic expression level of lincRNAs in relation to CRC initiation and progression has been addressed in many studies. For instance, Linc00284 was increased in CRC and was implicated in CRC progression. Linc00284 promoted the proliferation, migration, and invasion of CRC cells by acting as the sponge of miR-27a, and directly targeted c-Met via HGF/c-Met signaling (You et al., 2021). Other studies showed that Linc01578 facilitated the metastasis of colon cancer by forming a positive feedback loop with NF-κB/YY1 and suggested Linc01578 as a potential biomarker for prognosis and therapeutic target for colon cancer metastasis (Jia Liu et al., 2020). LINC00707, LINC00963, and LINC-RoR were found to be upregulated in CRC tissues and cells, yet LINC00312 was expressed at lower levels in CRC tissues and cells, introducing novel noncoding RNA-based diagnostics and therapeutics for colorectal malignancies (Li et al., 2018; Shao et al., 2019; Li et al., 2020; Wu et al., 2021). Liu et al. identified 144 lncRNAs differentially expressed in metastatic CRC patients compared with nonmetastatic CRC patients from The Cancer Genome Atlas (TCGA) database, and finally identified a 3-lncRNA signature (LINC00114, LINC00261, and HOTAIR) with the greatest prognostic value for CRC (Shuzhen Liu et al., 2020).

In our present study, we screened Linc01836, an uncharacterized lincRNA located on chromosome 19p13.3 *via* using TCGA database, which was markedly increased in CRC tissues. Moreover, to date, few studies about Linc01836 have been recorded; thus, the clinical significance of aberrant Linc01836 expression in CRC patients is still elusive. In our study, we sought to investigate the expression level of circulating Linc01836 and evaluate its value in the diagnosis, efficacy monitoring, and prognosis of CRC. Additionally, we analyzed the cell localization of Linc01836 in CRC cells and further explored the downstream ceRNA network that might be involved in CRC progression and prognosis.

## Materials and Methods

### Patients and Samples

Samples were collected from the Affiliated Hospital of Nantong University (Nantong, China) between February 2020 and June 2021, which were the remaining serum samples for routine tests. A total of 222 patients were recruited, including 171 patients clinically defined as CRC, 51 patients diagnosed as benign adenomas or adenomatous polyposis coli. CRC patients were diagnosed by histopathology, and tumors were staged and graded according to the 2017 AJCC/UICC TNM classification. A total of 138 control participants were recruited from a large crowd of individuals from the Health and Disease Management Center of the Affiliated Hospital of Nantong University. The demographic and clinical characteristics of researched patients and healthy folks are summarized in Table 1.This study was approved by the Human Research Ethical Committee of the Affiliated Hospital of Nantong University (Ethics Review Report No. 2018-L055), and the serum was stored at −80°C prior to further analysis.

**TABLE 1:** Demographic and clinical characterization of the study population.

Variables	Preoperational CRC (n = 137)	Postoperational CRC (n = 82)	Benign (*n* = 51)	Healthy (*n* = 138)
Age				
Median (range)	65 (36.88)	66 (40.80)	60 (29.78)	52 (39.84)
Gender				
Male	78	53	28	55
Female	59	29	23	83

### Bioinformatic Analysis

Gene expression data (HTSeq-FPKM) with clinical information of CRC samples (COAD/READ) were collected from TCGA (https://cancergenome.nih.gov/). The R Project for Statistical Computing (https://www.R-project.org) was applied to generate the lncRNA expression matrix. The overall survival data from TCGA database were also downloaded and analyzed. The software of RNAhirbd and RNAhybrid database (http://bibiserv.techfak.uni-bielefeld.de/rnahybrid/) were utilized for the exploration of downstream ceRNA network on the development and prognosis in CRC.

### RNA Extraction and Complementary DNA Synthesis

Serum RNA was extracted using Total RNA Fast Extraction Kit for Blood or Liquid Sample (BioTeke, Wuxi, China), and RNA in cells was extracted by TRIzol reagent. The nuclear and cytoplasmic fractions were isolated utilizing Ambion^®^ PARIS^™^ Kit (Thermo Scientific, United States). All the extraction procedures were strictly under the manufacturer’s protocol. The concentration and integrity of RNA samples were assured by NanoDrop spectrophotometer (Thermo Fisher Scientific, United States). The OD260/280 ratio between 1.8 and 2.0 indicated good purity, and RNA samples were maintained at −80°C before use.

Extracted RNA was reversely transcribed into cDNA by the RevertAid First Strand cDNA Synthesis Kit (Thermo Fisher Scientific, MA, United States) following the manufacturer’s protocol. The 20-μl reaction mixture was centrifuged briefly, followed by incubation at 42°C for 60 min and subsequently at 70°C for 5 min.

### Quantitative Real-Time Polymerase Chain Reaction

The relative expression level of Linc01836 was determined by qRT-PCR, which was conducted using the LightCycler 480 (Roche, Switzerland). The sequences of primers are in Table 2, and the PCR procedure was set as follows: 95°C for 30 s, followed by 45 cycles of 95°C for 5 s and 60°C for 30 s. ChamQ Universal SYBR qPCR Master Mix (Vazyme Biotech Co., Ltd.) was used as DNA-specific fluorescent dye, 18S rRNA was selected as a housekeeping gene, and the result for each sample was normalized to 18S rRNA expression. Relative expression was determined by the comparative Ct method (2^−ΔΔCt^), and all experiments were carried out in three replications.

**TABLE 2:** Primer sequences.

Primer	Sequence
18S rRNA	F: GTA​ACC​CGT​TGA​ACC​CCA​TT
	R: CCA​TCC​AAT​CGG​TAG​TAG​CG
Linc01836	F: CGA​GGA​GGA​CAA​GGG​AGG​GAA​C
	R: TGG​TGA​GCA​GGA​GGG​ACT​TAG​C

### Agarose Gel Electrophoresis

TAE solution and GelRed dye were used to prepare the 2% agarose gel. A mixture of 5 μl of PCR product and 1 μl of 6× loading buffer was loaded to the given position of solidified agarose gel, and electrophoresis was performed at a voltage of 120 V for 40 min. Images were obtained by a GelDoc XR Imaging System (Bio-rad, United States ).

### Detection of Other Tumor Markers

The serum CEA, CA19-9, Cyfra21-1, and carbohydrate antigen 72-4 (CA72-4) concentrations in CRC patients and healthy controls were measured by Architect I4000 SR (Abbott, Chicago, IL, United States ).

### Statistical Analysis

All data obtained from three or more independent experiments were displayed as mean ± standard deviation (SD). The software of SPSS 20.0 (IBM SPSS Statistics, Chicago, IL, United States) and GraphPad Prism 8.0 (GraphPad Software, La Jolla, CA, United States) were used for statistical analysis. Student’s *t*-test was performed to do the comparison between two groups when the dataset obeyed Gaussian’s distribution; otherwise, Mann–Whitney *U*-test could be used. One-way ANOVA was applied for comparison between more groups. The diagnostic value of Linc01836 and other tumor markers for differentiating CRC patients from the healthy folks was evaluated by receiver operating characteristic (ROC) curves. The relationship between serum Linc01836 expression and clinicopathological characteristics of CRC cases was analyzed *via* chi-square test. Survival analysis using data from TCGA database was performed. Values of *p* < 0.05 were deemed as statistically significant.

## Results

### The Identification of Linc01836

**FIGURE 1:**
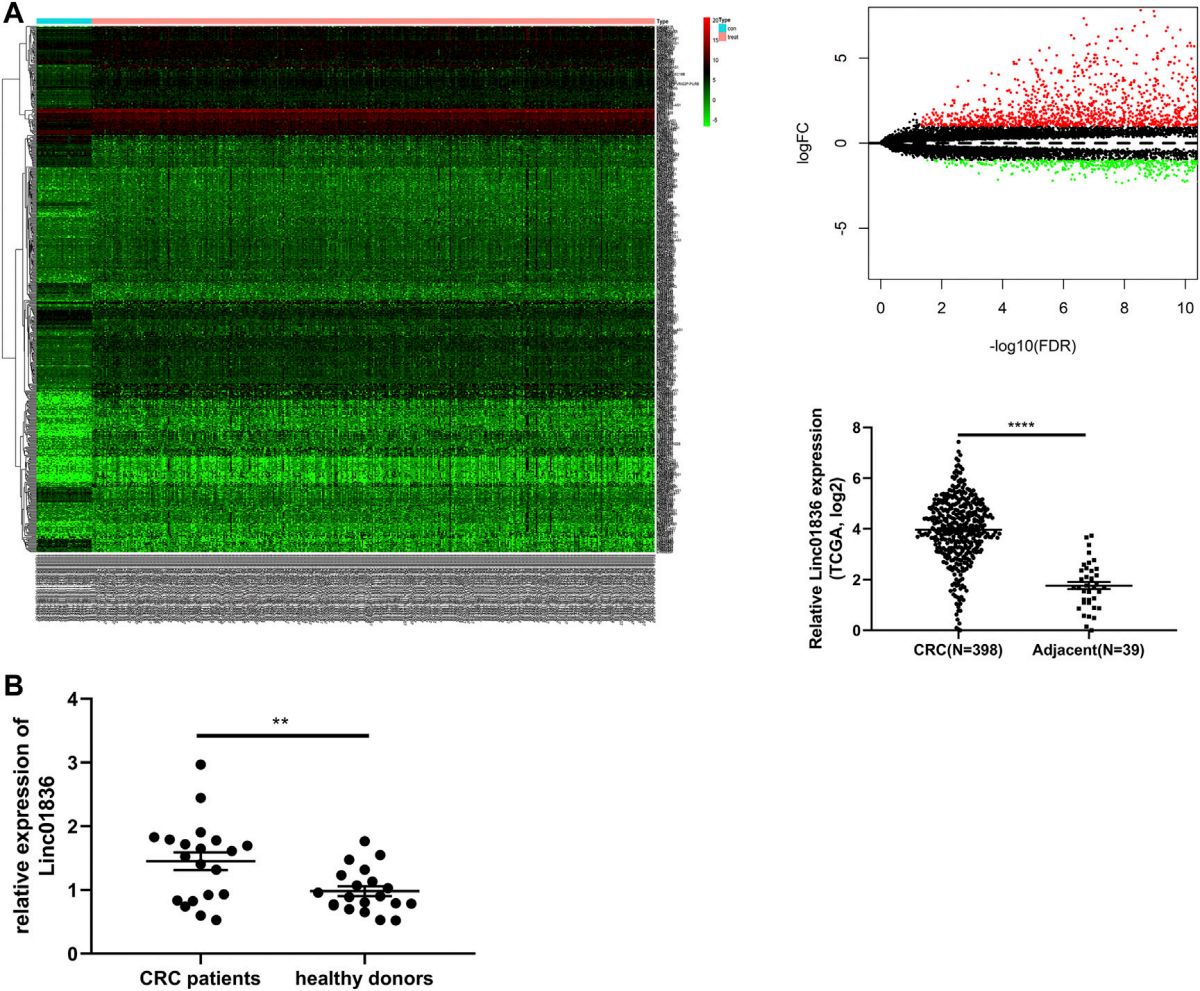
Identification of Linc01836. **(A)** The dysregulated expression level of Linc01836 in The Cancer Genome Atlas (TCGA) database. **(B)** The expression level of serum Linc01836 in CRC patients (*n* = 20) and healthy donors (*n* = 20). *p < 0.05, **p < 0.01, ***p < 0.001, ****p < 0.0001, NS *p* > 0.05.

CRC-related transcriptome sequencing data from TCGA database were downloaded and analyzed. A total of 599 lncRNAs were found to be differentially expressed between CRC tissues and adjacent normal tissues, including 472 upregulated and 127 downregulated. Linc01836 was highly expressed in CRC tissues (Figure 1A). Thereafter, we further analyzed the clinicopathological data and survival data of CRC patients from TCGA database, and found that upregulated Linc01836 was associated with short survival time and poor prognosis (Figure 3D). Analysis of qRT-PCR was primitively used to determine the serum expression level of Linc01836 in 20 CRC patients as well as in 20 healthy controls. The expression of Linc01836 was significantly upregulated in CRC (1.451 ± 0.628), compared with healthy samples (0.982 ± 0.341) (Figure 1B). The results indicated that circulating Linc01836 might increase in CRC patients.

### Methodology Evaluation of Serum Linc01836 Detection

**FIGURE 2:**
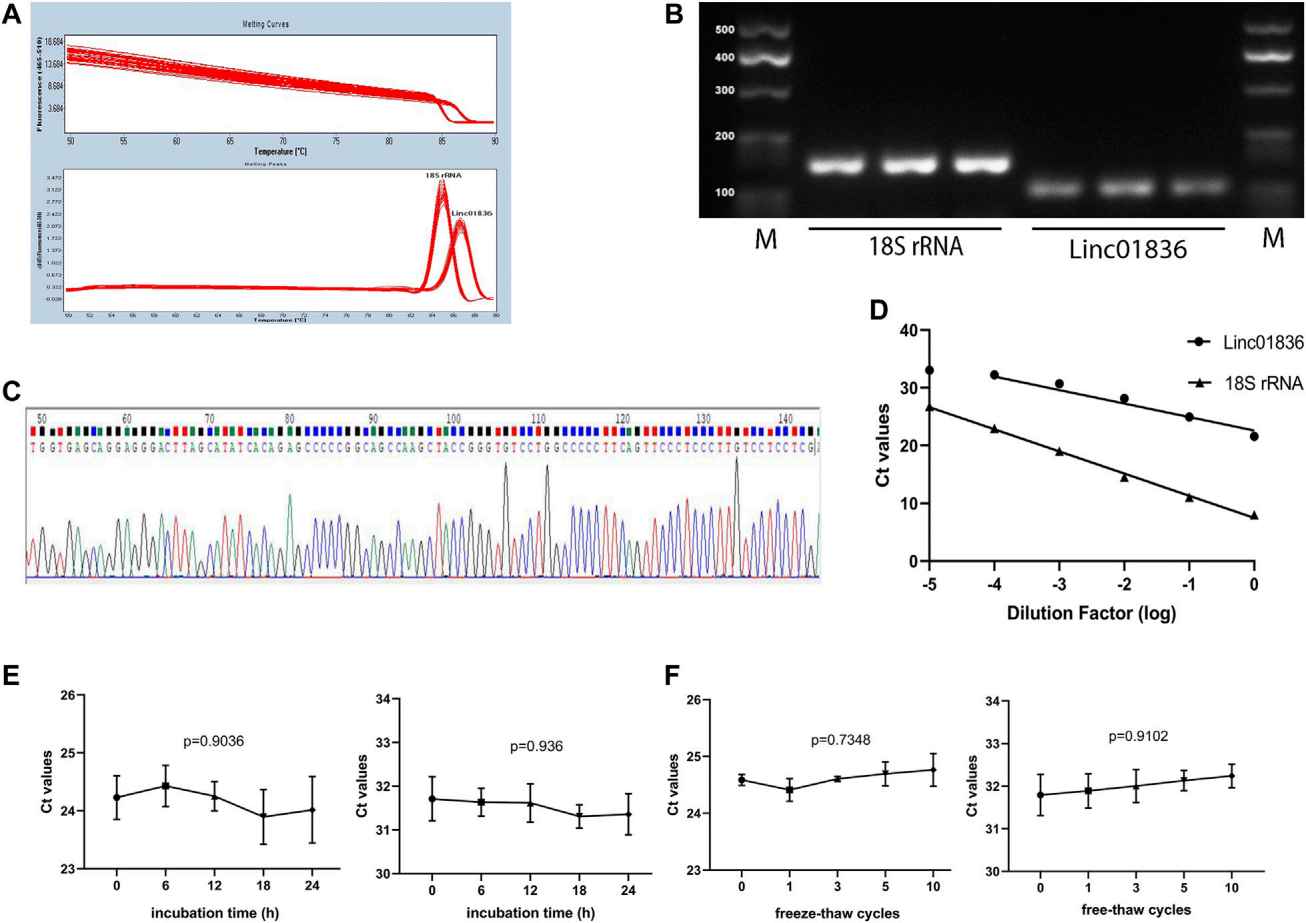
Methodological evaluation of Linc01836. **(A)** The melting peak of Linc01836 showed a single peak. **(B)** The qRT-PCR products detected by agarose gel electrophoresis exhibited a single band. **(C)** Sanger sequencing alignment of the amplification product was confirmed to be Linc01836. **(D)** The linearity of Linc01836 and 18S rRNA were acceptable. **(E, F)** The detection of 18S rRNA and Linc01836 at room temperature within 24 h and repeated freeze-thaw cycles showed good stability. *p < 0.05, **p < 0.01, ***p < 0.001, ****p < 0.0001, NS *p* > 0.05.

Given that there was no unified internal reference for serum lncRNA detection in CRC, 18S rRNA, and glyceraldehyde-3-phosphate dehydrogenase (GAPDH), two most commonly used reference genes were taken into consideration. We detected their expression in 12 mixed sera, and finally, 18S rRNA was chosen for the reference gene in later research, which showed lower Ct value and acceptable repeatability (Supplementary Table S1). To verify whether the self-established qRT-PCR method for the detection of Linc01836 was available for clinical laboratory analysis, methodology evaluation, including specificity, linearity, intra- and interassay imprecision and stability, was performed. Specific single peak was exhibited in the qRT-PCR melting curves (Figure 2A). A single band also appeared in the agarose gel electrophoresis of the PCR products (Figure 2B). Sequence alignment confirmed that the amplification product was exactly Linc01836 (Figure 2C). Serial dilutions (1:1, 1:10, 1:100, 1:1,000, 1:10,000, and 1:100,000) of cDNA from serum sample with high Linc01836 expression were used to assess the linearity. The regression equation of 18S rRNA was y = −3.832x + 7.486, and the R^2^ of the standard curve was 0.9970. Meanwhile, the regression equation of Linc01836 was y = −2.338x + 22.6, and the R^2^ of the standard curve was 0.9532. When expurgating the data from the maximum dilution factor (1:100,000), the R^2^ of the Linc01836 standard curve could be raised up to 0.9810, which meant that the qRT-PCR method we established exhibited good linearity and could be applicable for serum Linc01836 detection at a very low concentration (Figure 2D). For the evaluation of imprecision, intra-assay imprecision was evaluated by 12 aliquots of mixed serum within one experimental process, and the inter-assay imprecision was performed on 12 separate days using the same samples. Related data are listed in Table 3, suggesting satisfactory precision for clinical detection. Next, we investigated the stability of serum Linc01836 by both the room temperature incubation test and the freeze–thaw experiment. In the room temperature incubation test, serum aliquots were maintained for 0, 6, 12, 18, and 24 h at room temperature. In the freeze–thaw experiment, serum aliquots were frozen and thawed 0, 1, 3, 5, and 10 times. No significant changes were found for the expressions of Linc01836 and 18S rRNA (Figures 2E, F). Taken together, all these results indicated that the performance of the self-established qRT-PCR method for Linc01836 detection in serum was satisfactory and suitable for further research.

**TABLE 3:** Imprecision for Linc01836 and 18S rRNA (N = 12).

	RNA	Ct (mean ± SD)	CV, %
Intra-assay	18S rRNA	18.34 ± 0.35	1.91
	Linc01836	28.76 ± 0.17	0.60
Inter-assay	18S rRNA	17.76 ± 0.93	5.24
	Linc01836	28.58 ± 0.40	1.40

### Upregulated Serum Linc01836 in Colorectal Cancer Patients

**FIGURE 3:**
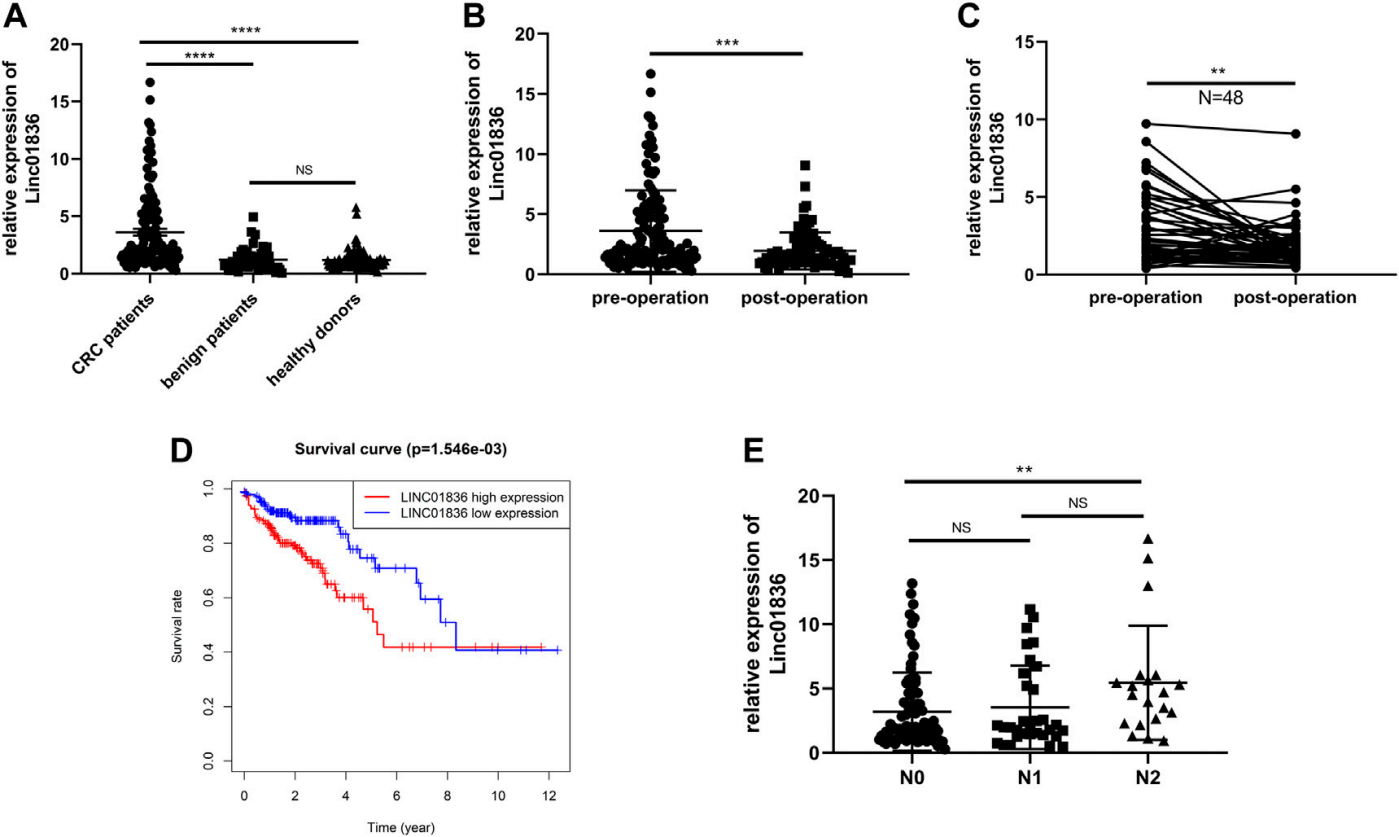
Serum Linc01836 serving as a biomarker for early diagnosis and cancer surveillance. **(A)** The expression level of serum Linc01836 in colorectal cancer (CRC) patients (*n* = 137), healthy donors (*n* = 138), and patients with benign colorectal diseases (*n* = 51). **(B)** The expression level of serum Linc01836 in preoperational CRC patients (n = 137) and postoperational CRC patients (*n* = 82). **(C)** The expression level of serum Linc01836 in 48 pairs of CRC patients before and after surgical resection. **(D)** Survival curve verified the prognostic value of Linc01836. **(E)** The expression level of serum Linc01836 in CRC patients with different stages of lymph node metastasis. *p < 0.05, **p < 0.01, ***p < 0.001, ****p < 0.0001, NS *p* > 0.05.

Serum Linc01836 expression levels in 137 CRC patients, 51 patients with benign adenomas or adenomatous polyposis coli, and 138 healthy donors were detected by the abovementioned qRT-PCR, and normalized to the reference gene 18S rRNA. Since serum Linc01836 expressions did not fit the normal distribution, data were shown in the format of median and 95% confidence limit (CI). It was found that serum Linc01836 expression in CRC patients, benign patients, and healthy donors were 2.208 (1.765, 2.567), 1.104 (0.714, 1.320), and 0.990 (0.901.1.110), respectively. As illustrated in Figure 3A, serum Linc01836 expression in CRC patients was significantly higher than that in the patients with benign colorectal diseases (*p* < 0.0001) and healthy controls (*p* < 0.0001), but there was no significant difference between benign and healthy groups (*p* = 0.4934). These results suggested that serum Linc01836 could be conducive in the auxiliary diagnosis of CRC.

According to previous studies, some circulating noncoding RNAs with upregulated expressions in tumor patients could return to a favorable level, some even showing no significance with expressions in healthy people. To verify whether detection of serum Linc01836 expression was helpful for tumor dynamic monitoring, a comparison of Linc01836 expressions between 137 preoperational sera and 82 postoperational sera was carried out (Figure 3B). Results manifested that the level of serum Linc01836 decreased significantly after surgical resection (*p* = 0.0001). Moreover, we analyzed circulating Linc01836 expressions from 48 pairs of CRC patients before and after surgery. As illustrated in Figure 3C, results from pre- and postoperation groups also confirmed the same conclusion that serum Linc01836 levels declined after surgery (*p* = 0.0082). Moreover, to better understand the relationship between overall survival rate and Linc01836 expression in CRC patients, we downloaded the survival data from TCGA database and classified CRC patients into high-expression group and low-expression group by the median of Linc01836 expression in tissues. The survival curve showed that the high-expression group possessed lower overall survival rate (Figure 3D). To sum up, serum Linc01836 could be served as a new tumor marker for early diagnosis of CRC and might be used for cancer surveillance.

### Relevance Between Linc01836 Expression and Clinicopathological Characteristics

To further understand the clinical application of serum Linc01836 detection, the clinicopathological features of the 137 CRC patients are summarized in Table 4. CRC patients were divided into two groups according to the median (2.028) of serum Linc01836 expression: relative high group (expression >2.028, *n* = 68) and relative low group (expression ≤2.028, *n* = 69). Chi-square test showed that high serum Linc01836 expression levels were significantly associated with histological stage (*p* = 0.002) and lymph node metastasis (*p* = 0.006), but had no statistical significance with other parameters, such as gender (*p* = 0.349), age (*p* = 0.932), tumor size (*p* = 0.797), tumor location (*p* = 0.671), nerve/vascular invasion (*p* = 0.917), and morphological characteristics (*p* = 0.808). It was worthy to mention that the T stage represented the depth of tumor invasion; even though no statistical significance was found in the T stage and TNM stage in general, it displayed obvious correlation between increased serum Linc01836 expression and lymph node metastasis. We then compared the serum Linc01836 expression in 137 CRC patients at different stages of lymph node metastasis, and the results manifested a significant difference between N0 and N2 stages (*p* = 0.0144). Although the difference between N0 and N1 stages (*p* = 0.621) and the difference between N1 and N2 stages (*p* = 0.113) did not show any significance, we still could intuitively see that serum Linc01836 levels increased gradually with the escalating degrees of lymph node metastasis (Figure 3E).

**TABLE 4:** Correlation between Linc01836 expression and clinicopathologic parameters of colorectal cancer (CRC) patients.

Characteristics	No. of patients	Linc01836 expression		*p*-Value
		Low	High	
Gender				
Male	78	42	36	0.349
Female	59	27	32	
Age				
≤65	69	35	34	0.932
>65	68	34	34	
Tumor size, cm				
<4	69	34	35	0.797
≥4	68	35	33	
Histological stage				
Poor	20	5	15	0.002
Moderate	100	50	50	
Well	17	14	3	
T stage				
T1–T2	46	24	22	0.763
T3–T4	91	45	46	
Lymph node metastasis				
Yes	49	17	32	0.006
No	88	52	36	
Nerve/vascular invasion				
Positive	55	28	27	0.917
Negative	82	41	41	
Location				
Colon	71	37	34	0.671
Rectum	66	32	34	
Morphological characteristic				
Ulcerative	98	50	48	0.808
Protrusive	39	19	20	
TNM stage				
I–II	78	44	34	0.104
III–IV	59	25	34	

### Assessment of Diagnostic Value of Linc01836 and Other Commonly Used Tumor Biomarkers for Colorectal Cancer

**FIGURE 4:**
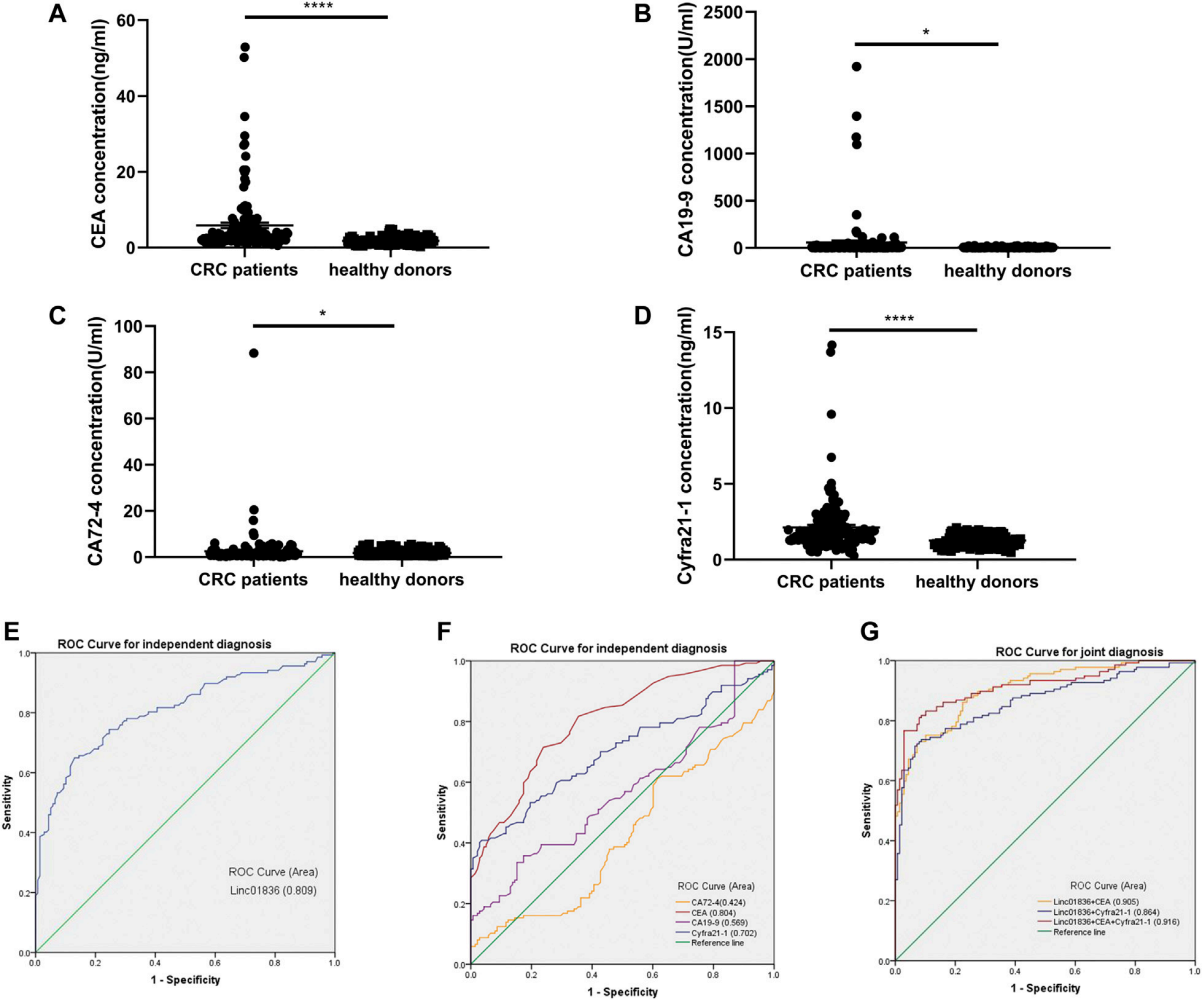
Diagnostic value of Linc01836 and other commonly used tumor biomarkers for CRC. **(A–D)** The expression levels of serum CEA, CA19-9, CA72-4, and Cyfra21-1 in CRC patients (*n* = 137) and healthy donors (*n* = 138) **(E,F)** Receiver operating characteristic (ROC) curve analysis of Linc01836, CEA, CA19-9, CA72-4 ,and Cyfra21-1 in independent diagnosis of CRC patients and healthy donors. **(G)** ROC curve analysis of Linc01836, CEA, and Cyfra21-1 in joint diagnosis of CRC patients and healthy donors. *p < 0.05, **p < 0.01, ***p < 0.001, ****p < 0.0001, NS *p* > 0.05.

Although the sensitivity and specificity of commonly used serum tumor biomarkers were undesirable, they still were most widely used in clinics for the early screening of malignancies. Therefore, we also examined serum CEA, CA19-9, Cyfra21-1, and CA72-4 levels in CRC patients and healthy donors. Results are shown in Supplementary Table S2, exhibiting higher concentrations in the CRC group (Figures 4A–D). ROC curves were generated to assess the potential of Linc01836 along with these serum tumor biomarkers as diagnostic biomarkers for CRC, which demonstrated that serum Linc01836 could effectively differentiate CRC patients from the healthy folks, with the favorable area under the curve (AUC) of 0.809 (95% CI: 0.757–0.861, *p* < 0.001, Figure 4E). CEA, as the most commonly used tumor biomarker in the clinic, exhibited similar diagnostic value, with an AUC of 0.804 (95% CI: 0.754–0.855, *p* < 0.001). Cyfra21-1 was usually used for the diagnosis of lung cancer, non-small cell lung cancer (NSCLC), in particular. Surprisingly, it also possessed acceptable diagnostic value, with an AUC of 0.702 (95% CI: 0.639–0.764, *p* < 0.001) in our research. However, CA19-9 and CA72-4, as commonly accepted gastrointestinal tumor markers, did not perform well with AUCs of 0.569 (95% CI: 0.500–0.637, *p* = 0.049) and 0.424 (95% CI: 0.356–0.492, *p* = 0.030), respectively (Figure 4F). Cutoff value was often set by the expression level at the maximum value of the Youden index. As for the relative Linc01836 expression in serum samples, the cutoff value was defined as 1.63, offering sensitivity of 65.0% and specificity of 87.0%, and the accuracy of differentiating CRC from a healthy population could reach 76.0%, which was superior to CEA (73.8%), Cyfra21-1 (68.7%), CA19-9 (59.3%), and CA72-4 (53.1%). Subsequently, we drew the ROC curves for the joint diagnosis and evaluated their efficacy concomitantly. As illustrated in Figure 4G and Table 5, combined detection of Linc01836, CEA, and Cyfra21-1 displayed the best diagnostic efficacy, harboring an AUC of 0.916 (95% CI: 0.881–0.950, *p* < 0.001), higher than that of a combination of Linc01836 and CEA [AUC = 0.905 (95% CI: 0.871–0.939, *p* < 0.001)] and a combination of Linc01836 and Cyfra21-1 [AUC = 0.864 (95% CI: 0.820–0.909, *p* < 0.001)]. Our results confirmed the diagnostic utility of serum Linc01836, CEA, and Cyfra21-1 and their combination in differentiating CRC patients from healthy population.

**TABLE 5:** Independent or joint diagnostic value of Linc01836 and other commonly used tumor biomarkers for distinguishing CRC patients from healthy population.

	SEN, %	SPE, %	ACCU, %	PPV, %	NPV, %
Linc01836	65.0 (89/137)	87.0 (120/138)	76.0 (209/275)	83.2 (89/107)	71.4 (120/168)
CEA	71.5 (98/137)	76.1 (105/138)	73.8 (203/275)	74.8 (98/131)	72.9 (105/144)
Cyfra21-1	40.9 (56/137)	96.4 (133/138)	68.7 (189/275)	91.8 (56/61)	62.1 (133/214)
CA19-9	35.8 (49/137)	82.6 (114/138)	59.3 (163/275)	67.1 (49/73)	56.4 (114/202)
CA72-4	8.8 (12/137)	97.1 (134/138)	53.1 (146/275)	75.0 (12/16)	51.7 (134/259)
Linc01836 + CEA	90.5 (124/137)	65.2 (90/138)	77.8 (214/275)	72.1 (124/172)	87.4 (90/103)
Linc01836 + Cyfra21-1	81.0 (111/137)	83.3 (115/138)	82.2 (226/275)	82.8 (111/134)	81.6 (115/141)
Linc01836 + CEA + Cyfra21-1	92.0 (126/137)	62.3 (86/138)	77.1 (212/275)	70.8 (126/178)	88.7 (86/97)

Note. Sensitivity (SEN), specificity (SPE), overall accuracy (ACCU), positive predictive value (PPV), and negative value (NPV).

### Exploration of the Downstream Regulatory ceRNA Network of Linc01836 in Colorectal Cancer

**FIGURE 5:**
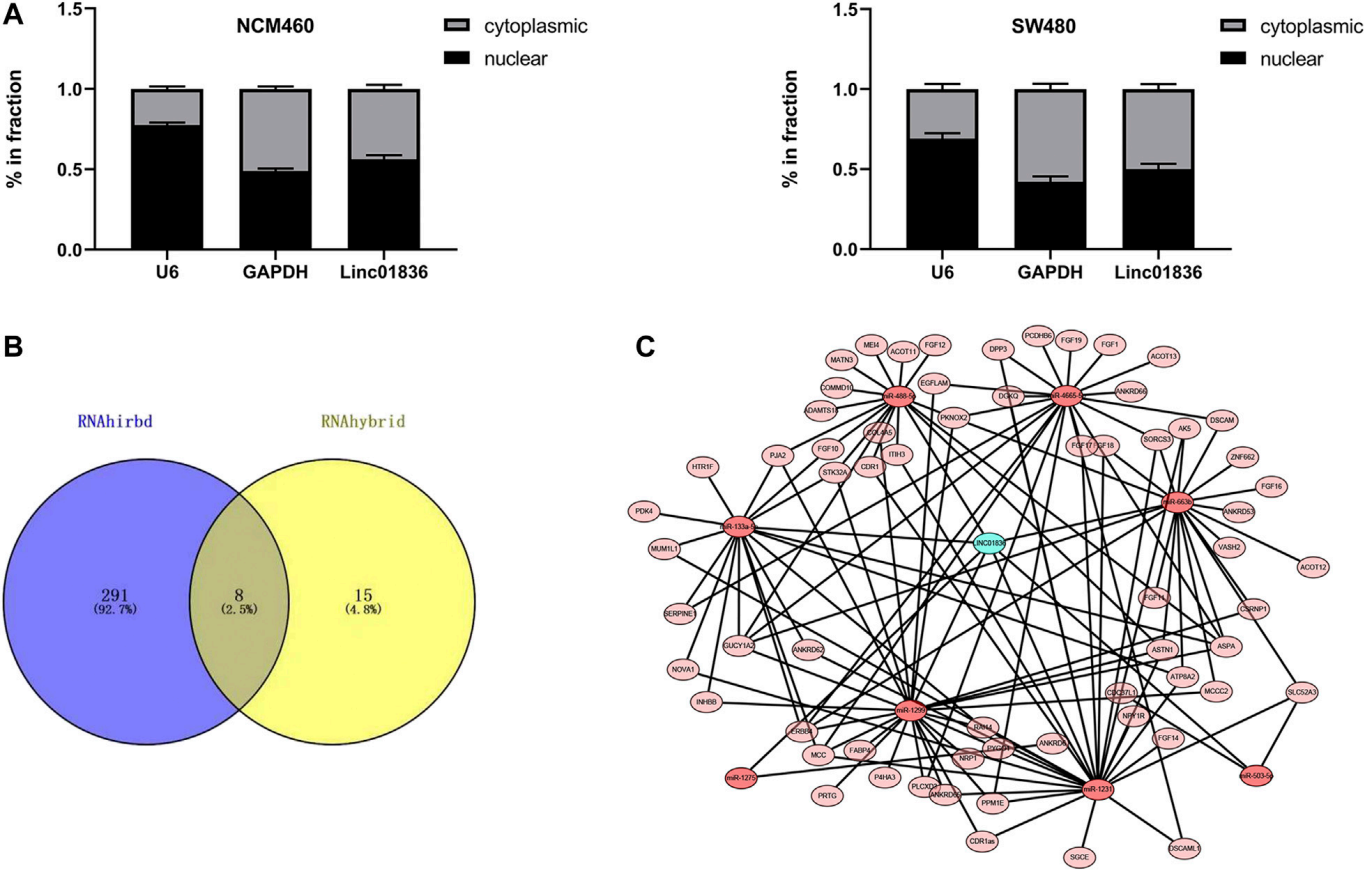
Exploration of the downstream regulatory ceRNA network of Linc01836 in CRC. **(A)** Detection of Linc01836 localization in NCM 460 and SW480 cells by nuclear and cytoplasmic RNA separation assay. **(B)** Schematic illustration exhibiting the overlap between the target miRNAs predicted by RNAhirbd and RNAhybrid database. **(C)** Prediction of lncRNAs–miRNAs–mRNAs network map of Linc01836.

To investigate the biological function and underlying mechanisms, we extracted RNA from NCM460 and SW480 cells by nucleoplasm separation, and qRT-PCR results manifested that Linc01836 was averagely located in the nucleus and cytoplasm, suggesting that it might participate in CRC progression through both transcriptional and posttranscriptional regulation (Figure 5A). Crosstalk among lncRNAs, miRNAs, and mRNAs plays a pivotal role in the initiation and progression of CRC (Wang et al., 2019; Xu et al., 2019). Subsequently, the potential lincRNA–miRNA–mRNA regulatory axis in CRC was predicted by bioinformatics analysis. As shown in Figures 5B, C, eight miRNAs (hsa-miR-663b, hsa-miR-503-5p, hsa-miR-1231, hsa-miR-4665-5p, hsa-miR-1299, hsa-miR-133a-5p, hsa-miR-488-5p, and hsa-miR-1275) and their corresponding target mRNAs associated with prognosis were depicted, which might offer a new guidance in exploring the regulatory network of Linc01836 in CRC in the future.

## Discussion

Colorectal cancer (CRC) is a common carcinoma of the gastrointestinal tract originating from either the colon or the rectum, and the pathogenesis remains largely unknown (Yang et al., 2016; Wang et al., 2019). Accumulating evidence has shown that lncRNAs play critical roles in the initiation and progression of CRC. Some lncRNAs can be released into the blood as biomarkers for tumor diagnosis (Meng et al., 2021; Sun et al., 2021). Yin et al. reported that lncRNA NNT-AS1 was upregulated in the serum of CRC patients, and the expression levels of NNT-AS1 were significantly decreased in postoperative samples compared with preoperative samples (Esfandi et al., 2019). It was demonstrated that serum lncRNA B3GALT5-AS1 was significantly decreased in CRC. In addition, increasing serum B3GALT5-AS1 level was related to histological differentiation and TNM stage. Therefore, B3GALT5-AS1 might be a novel potential diagnostic biomarker for CRC (Ding et al., 2020). Our preliminary experiment confirmed that Linc01836 was significantly higher in the serum of CRC than in healthy folks. Moreover, Linc01836 is an uncharacterized and hardly reported lncRNA, so we finally chose Linc01836 for the following study.

In our study, serum Linc01836 expression in CRC patients was markedly higher than those in healthy samples. We screened out this upregulated lncRNA in CRC patients through the data from TCGA database. In the present study, the expression of serum Linc01836 was determined by the qRT-PCR method, which was verified to be specific, accurate, and reproducible beforehand. Results showed that Linc01836 expressions in CRC patients were significantly higher than those in patients with benign colorectal diseases and healthy folks, and there was no significant difference between benign and healthy groups, suggesting the potential of serum Linc01836 detection in the auxiliary diagnosis of CRC. Subsequently, we analyzed the relevance between Linc01836 and clinicopathological parameters, and discovered that a high level of serum Linc01836 was associated with lymph node metastasis and histological stage, but had no statistical significance with other parameters including gender, age, tumor size, depth of tumor invasion, nerve/vascular invasion, tumor location, and morphological characteristics. We then compared the serum Linc01836 expression in CRC patients at different stages of lymph node metastasis, and the results manifested a significant difference between the N0 and N2 stages. Moreover, to verify whether serum Linc01836 performed better than other common tumor markers in the early detection of CRC or screening, we compared the diagnostic efficacy of Linc01836 and the routinely used CEA, CA19-9, Cyfra21-1, and CA72-4 by ROC curves. The results demonstrated that the detection of Linc01836 alone could distinguish CRC patients from healthy individuals with acceptable sensitivity and specificity, with AUC even bigger than the other four markers, confirming the promising clinical application of serum Linc01836 as a novel and effective biomarker for CRC (Lech et al., 2016; Wilhelmsen et al., 2017). Besides, the combined detection of CEA or Cyfra21-1 and Linc01836 both improved the diagnostic accuracy. Moreover, an algorithm based on the combination of CEA, Cyfra21-1, and Linc01836 exhibited the best diagnostic efficacy. In addition, Linc01836 expression in serum was found to significantly decline after surgical resection. Furthermore, the survival data from TCGA database confirmed that a higher level of serum Linc01836 was associated with lower overall survival rate of CRC patients, implying the ability of Linc01836 in cancer dynamic surveillance. Additionally, nucleocytoplasmic isolation assay confirmed that Linc01836 was averagely located in the nucleus and cytoplasm, suggesting that it might participate in CRC progression and prognosis through the crosstalk among lncRNAs, miRNAs, and mRNAs.

As was shown in our study, there were many limits. Most obviously, the sample size is relatively small. The real hardship lies in two major aspects: 1) It is difficult to recruit many CRC patients who are willing to draw blood before and several days after surgical resection. 2) It is even harder to exsanguinate when tumor relapses, which usually takes several years or even decades. To comprehensively analyze the application value of serum Linc01836 in early diagnosis and clinical dynamic surveillance of CRC, further identification is still needed. Since our results are just an exploratory single-center analysis, an independent multicenter validation is still needed. Besides, the predicted Linc01836–miRNAs–mRNAs regulatory axis only plays a crucial part in the cytoplasm; the regulatory mechanism in the nucleus is still illusive. Furthermore, future clinical and functional studies should be performed to precisely determine and verify the predicted regulatory mechanisms of Linc01836 in CRC initiation, progression, and prognosis.

To sum up, serum Linc01836 could be reliably and readily detected, ensuring its promising application prospect as a novel, valuable, and noninvasive biomarker for early detection, clinical surveillance, and prognosis of CRC. Moreover, the predicted crosstalk between lncRNAs, miRNAs, and mRNAs might offer a new guidance in exploring the regulatory network of Linc01836 in CRC development in the future.

## Data Availability

The original contributions presented in the study are included in the article/Supplementary Material, further inquiries can be directed to the corresponding authors.

## Abbreviations

(CA19-9), carbohydrate antigen 19; (CA50), carbohydrate antigen 50; (CA72-4), carbohydrate antigen 72-4; (CEA), carcinoembryonic antigen; (CI), confidence limit; (CRC), colorectal cancer; (GAPDH), glyceraldehyde-3-phosphate dehydrogenase; (lncRNA), long noncoding RNA; (ncRNA), noncoding RNA; (NSCLC), non-small cell lung cancer; (lincRNA), long intergenic noncoding RNA; (qRT-PCR), quantitative real-time polymerase chain reaction; (ROC) curves, receiver operating characteristic; (SD), standard deviation; (TCGA), The Cancer Genome Atlas.

## References

[B1] Chao J. Y. Chang H. C. Jiang J. K. Yang C. Y. Chen F. H. Lai Y. L. (2021). Using Bioinformatics Approaches to Investigate Driver Genes and Identify BCL7A as a Prognostic Gene in Colorectal Cancer. Comput. Struct. Biotechnol. J. 19, 3922–3929. 10.1016/j.csbj.2021.06.044 34306573 PMC8280477

[B2] Danese E. Montagnana M. Lippi G. (2019). Circulating Molecular Biomarkers for Screening or Early Diagnosis of Colorectal Cancer: Which Is Ready for Prime Time? Ann. Transl Med. 7, 610. 10.21037/atm.2019.08.97 32047771 PMC7011594

[B3] Ding Y. Feng W. Ge J. K. Dai L. Liu T. T. Hua X. Y. (2020). Serum Level of Long Noncoding RNA B3GALT5-AS1 as a Diagnostic Biomarker of Colorectal Cancer. Future Oncol. 16, 827–835. 10.2217/fon-2019-0820 32207329

[B4] Esfandi F. Taheri M. Kahaei M. S. Omrani M. D. Kholghi Oskooei V. Ghafouri-Fard S. (2019). Downregulation of Nicotinamide Nucleotide Transhydrogenase and its Naturally Occurring Antisense RNA in Gastric Cancer. Asia Pac. J. Clin. Oncol. 15, e191–e196. 10.1111/ajco.13230 31309731

[B5] Liu J. Zhan Y. Wang J. Wang J. Guo J. Kong D. (2020). Long Noncoding RNA LINC01578 Drives colon Cancer Metastasis through a Positive Feedback Loop with the NF-Κb/yy1 axis. Mol. Oncol. 14, 3211–3233. 10.1002/1878-0261.12819 33040438 PMC7718957

[B6] Lech G. Słotwiński R. Słodkowski M. Krasnodębski I. W. (2016). Colorectal Cancer Tumour Markers and Biomarkers: Recent Therapeutic Advances. World J. Gastroenterol. 22, 1745–1755. 10.3748/wjg.v22.i5.1745 26855534 PMC4724606

[B7] Li G. Wang C. Wang Y. Xu B. Zhang W. (2018). LINC00312 Represses Proliferation and Metastasis of Colorectal Cancer Cells by Regulation of miR-21. J. Cel Mol Med 22, 5565–5572. 10.1111/jcmm.13830 PMC6201213 30134003

[B8] Li X. Chen W. Jia J. You Z. Hu C. Zhuang Y. (2020). The Long Non-coding RNA-RoR Promotes the Tumorigenesis of Human Colorectal Cancer by Targeting miR-6833-3p through SMC4. Onco Targets Ther. 13, 2573–2581. 10.2147/OTT.S238947 32273727 PMC7109305

[B9] Mattiuzzi C. Sanchis-Gomar F. Lippi G. (2019). Concise Update on Colorectal Cancer Epidemiology. Ann. Transl Med. 7, 609. 10.21037/atm.2019.07.91 32047770 PMC7011596

[B10] Meng S. Dolo P. R. Guo P. Hong J. Li C. Zhu X. (2021). Expression of Long Non-coding RNA LINC01279 in Gastric Adenocarcinoma and its Clinical Significance. Asian J. Surg S1015-9584 (21), 00533–9. 10.1016/j.asjsur.2021.08.031 34507839

[B11] Peng W. X. Koirala P. Mo Y. Y. (2017). LncRNA-mediated Regulation of Cell Signaling in Cancer. Oncogene 36, 5661–5667. 10.1038/onc.2017.184 28604750 PMC6450570

[B12] Shao H. J. Li Q. Shi T. Zhang G. Z. Shao F. (2019). LINC00707 Promotes Cell Proliferation and Invasion of Colorectal Cancer via miR-206/FMNL2 axis. Eur. Rev. Med. Pharmacol. Sci. 23, 3749–3759. 10.26355/eurrev_201905_17801 31115001

[B13] Liu S. Cao Q. An G. Yan B. Lei L. (2020). Identification of the 3-lncRNA Signature as a Prognostic Biomarker for Colorectal Cancer. Int. J. Mol. Sci. 21, 9359. 10.3390/ijms21249359 PMC7764807 33302562

[B14] Siegel R. L. Miller K. D. Fuchs H. E. Jemal A. (2021). Cancer Statistics, 2021. CA A. Cancer J. Clin. 71, 7–33. 10.3322/caac.21654 33433946

[B15] Sun Y. Jing Y. Zhang Y. (2021). Serum lncRNA-ANRIL and SOX9 Expression Levels in Glioma Patients and Their Relationship with Poor Prognosis. World J. Surg. Onc 19, 287. 10.1186/s12957-021-02392-2 PMC8461887 34556140

[B16] Wang L. Cho K. B. Li Y. Tao G. Xie Z. Guo B. (2019). Long Noncoding RNA (lncRNA)-Mediated Competing Endogenous RNA Networks Provide Novel Potential Biomarkers and Therapeutic Targets for Colorectal Cancer. Int. J. Mol. Sci. 20, 5758. 10.3390/ijms20225758 PMC6888455 31744051

[B17] Wilhelmsen M. Christensen I. J. Rasmussen L. Jørgensen L. N. Madsen M. R. Vilandt J. (2017). Detection of Colorectal Neoplasia: Combination of Eight Blood-Based, Cancer-Associated Protein Biomarkers. Int. J. Cancer 140, 1436–1446. 10.1002/ijc.30558 27935033

[B18] Wu Y. Cong L. Chen W. Wang X. Qiu F. (2021). lncRNA LINC00963 Downregulation Regulates Colorectal Cancer Tumorigenesis and Progression via the miR-10b/FGF13 axis. Mol. Med. Rep. 23, 211. 10.3892/mmr.2021.11850 33495804 PMC7830939

[B19] Xu M. Chen X. Lin K. Zeng K. Liu X. Pan B. (2018). The Long Noncoding RNA SNHG1 Regulates Colorectal Cancer Cell Growth through Interactions with EZH2 and miR-154-5p. Mol. Cancer 17, 141. 10.1186/s12943-018-0894-x 30266084 PMC6162892

[B20] Xu M. Chen X. Lin K. Zeng K. Liu X. Xu X. (2019). LncRNA SNHG6 Regulates EZH2 Expression by Sponging miR-26a/b and miR-214 in Colorectal Cancer. J. Hematol. Oncol. 12, 3. 10.1186/s13045-018-0690-5 30626446 PMC6327409

[B21] Yang Y. Du Y. Liu X. Cho W. C. (2016). Involvement of Non-coding RNAs in the Signaling Pathways of Colorectal Cancer. Adv. Exp. Med. Biol. 937, 19–51. 10.1007/978-3-319-42059-2_2 27573893

[B22] You J. Li J. Ke C. Xiao Y. Lu C. Huang F. (2021). Oncogenic Long Intervening Noncoding RNA Linc00284 Promotes C-Met Expression by Sponging miR-27a in Colorectal Cancer. Oncogene 40, 4151–4166. 10.1038/s41388-021-01839-w 34050266 PMC8211564

[B23] Yu B. Ye X. Du Q. Zhu B. Zhai Q. Li X. X. (2017). The Long Non-coding RNA CRNDE Promotes Colorectal Carcinoma Progression by Competitively Binding miR-217 with TCF7L2 and Enhancing the Wnt/β-Catenin Signaling Pathway. Cell Physiol Biochem 41, 2489–2502. 10.1159/000475941 28472810

