# Supplementary material for: Upregulated Linc01836 in Serum Promisingly Serving as a Diagnostic and Prognostic Biomarker for Colorectal Cancer
Source: Front Pharmacol. 2022 Mar 18;13:840391. doi: 10.3389/fphar.2022.840391 (PMC8975208; doi:10.3389/fphar.2022.840391)
Supplement: Supplementary file 1 [file DataSheet1.docx]

# Supplementary Tables

**Table S1. Ct Values for candidate reference genes**

| Reference Genes | Ct (Mean ± SD) | CV, % |
| --- | --- | --- |
| 18S rRNA | 18.34±0.35 | 1.91 |
| GAPDH | 23.88±0.25 | 1.03 |

**Table S2. Concentrations of serum CEA, CA19-9, CA72-4 and Cyfra21-1**

| Biomarkers | CRC Group  (n=137) | Healthy Group  (n=138) | p value |
| --- | --- | --- | --- |
| CEA | 3.00 (2.60,3.60) | 1.55 (1.40,1.70) | ＜0.0001 |
| CA19-9 | 6.10 (5.10,7.40) | 5.40 (4.76,6.20) | 0.0493 |
| CA72-4 | 1.10 (1.03,1.36) | 1.44 (1.11,1.69) | 0.0298 |
| Cyfra21-1 | 1.67 (1.53,1.86) | 1.26 (1.13,1.35) | ＜0.0001 |
